# Inequality and fiscal policies in Uruguay by race

**DOI:** 10.1007/s10888-017-9373-7

**Published:** 2018-01-24

**Authors:** Marisa Bucheli, Maximo Rossi, Florencia Amábile

**Affiliations:** 1 Universidad de la Republica, Montevideo, Uruguay

**Keywords:** Inequality, Poverty, Race, Fiscal policy, Direct transfers

## Abstract

The aim of this study is to analyze the effect of fiscal policy by race, disaggregating to consider Uruguayans with primarily European, African and indigenous ancestry. We perform an incidence analysis, an estimation of the effect of fiscal policy on the poverty exit rate and an assessment of the impact on the average ethno-racial gaps. The findings support the idea that fiscal policy reduces (but does not eliminate) ethnic gaps. This result is led by health care and educational transfers, and to a lesser degree by direct transfers. We do not consider quality issues with public services, which may affect the estimated narrowing of gaps. Finally, we find that Afro-descendants and indigenous individuals do not capture the full potential of education transfers because of their high drop-out rate.

## 1 Introduction

Latin America has a racially and ethnically heterogeneous population composed mainly of indigenous people, whites (European ancestry) and Afro-descendant people. Evidence shows the presence of ethno-racial gaps in many socioeconomic areas such as poverty, income, living conditions, educational levels and labor market outcomes (Gandelman et al. 2011; Ferreira and Guignoux 2011; Bailey et al. 2014; Busso et al. 2005; Telles et al. 2015).

The Uruguayan population is more homogenous in racial and ethnic terms than those of most Latin American countries. According to Frega et al. (2008), between 1786 and 1812 at least 60,000 slaves were brought to Río de la Plata from Africa. In 1819, Afro-descendants were 25% of the population of Montevideo (Frega et al. 2005). At the end of the 19th century, the indigenous proportion of the population was very low due to wars, disease and campaigns of extermination of groups that populated the territory before independence (Bracco 2004). The migration process during the 19th and the first half of the 20th century, mostly of European people, additionally reduced the proportion of Afro-descendant and indigenous people in the total population (Cabella and Nathan 2014).

Currently, Uruguay’s population is predominantly composed of Spanish and Italian descendants who self-classify as white in national censuses and surveys. Minorities account for a small portion of the population; according to the last census, less than 5% of people self-identified as mainly African and 2% indicated being predominantly of indigenous descent.

Perhaps due to this population structure, Uruguayan society had accepted the belief that there was not inequality or barriers to social mobility based on race. This self-image was reinforced by the fact that minorities share with the whole population language, values and cultural traits. Additionally, the long tradition of social policies and the absence of official racism supported the impression that there has been equality of opportunities and that minorities do not have their own specific problems. In this context, racial issues were not relevant in political agendas or in academic social research until recently. In the last decade, this perspective changed mainly because of pressure from minority organizations, international agencies and a small elite of Afro-descendant intellectuals and activists. In fact, recent studies of Afro-descendant and indigenous populations in Uruguay show that ethno-racial gaps in education, discrimination in the labor market and poverty all exist (Bucheli and Cabella 2010; Gonzalez and Sanroman 2010; Bucheli and Porzecanski 2011; Cabella and Nathan 2014).

The aim of this paper is to analyze the effect of taxes, direct transfers (in-cash benefits and food transfers), health care (public provision and private-sector subsidies) and public educational services on closing ethno-racial gaps. We distinguish three ethno-racial groups: whites, Afro-descendants and indigenous people.

Several studies of Latin America show that fiscal policy has an equalizing effect mainly due to public transfers—not tax policy—and due to the reduction of inequalities in the labor market (Breceda et al. 2009; Lustig et al. 2014a; Goñi et al. 2011); public transfers also contribute to poverty reduction (Alvaredo and Gasparini 2015). The same is true in Uruguay (Amarante et al. 2012; Bucheli et al. 2014), where public policies play an important role in ensuring low levels of inequality and poverty relative to Latin American standards (Lustig et al. 2014a). Latin American studies that differentiate the effects of fiscal policy by race are scarce (Cabrera et al. 2015; Pereira 2016; Lustig 2014b) and our study contributes to filling this gap in the literature.

Another strength of our study is the consideration of in-kind transfers, specifically education and health care. Studies on the effect of fiscal policy often focus exclusively on cash transfers. However, the importance of subsidies for and public provision of education and health care is two-fold: they are provided for redistributive purposes and they are usually a higher proportion of public spending than in-cash transfers. Moreover, there is evidence that these in-kind dimensions reduce inequality in Latin America, with a more sizeable effect than the tax and monetary benefits system (Breceda et al. 2009; Sahn and Younger 2006). The same is observed in Uruguay (Bucheli et al. 2014).

Our work contributes to the literature in three main ways: a) an incidence analysis showing the share of each ethno-racial group in transfers received and in taxes paid, b) an assessment of the effect of direct transfers on exiting poverty and c) a study of the impact of transfers and taxes on average income gaps. In Uruguay, policies do not distinguish the targeted population by race. Broadly speaking, direct transfer programs focus on low-income populations whereas in-kind transfers (education and health) are related to age. So, the effect of these policies on ethno-racial gaps responds to factors such as ethno-racial differences in income before taxes and transfers, demographic characteristics and program uptake. Although we do not aim to disentangle these factors in this paper, we discuss their importance.

This paper is structured in the following manner. In Section 2 we describe the database, income variables and racial classification utilized in this study. In Section 3 we present a brief description of welfare by racial group using disposable income. In Section 4 we analyze the effects of fiscal policy, and Section 5 concludes.

## 2 Data

We use the so-called Commitment to Equity (CEQ) database^1^. This database was constructed from Uruguay’s 2009 household survey, the Encuesta Continua de Hogares or ECH, collected by the National Institute of Statistics (Instituto Nacional de Estadística or INE 2009). This survey contains 130,054 observations of individuals in 46,936 households.

The relevant variables for our analysis are taxes, benefits and five concepts of income measured at the household level and considered in per capita terms. The five income concepts are defined by Lustig and Higgins (2013), and a detailed account of the procedures for estimating them for Uruguay can be found in Bucheli et al. (2012).

In this paper the unit of analysis is the individual, and the variables analyzed (income, taxes and benefits) are in household per capita terms. In the remainder of this section we present a brief review of these variables, the poverty lines used in the analysis and a description of the population.

### 2.1 Taxes, public benefits and income variables

Market income is defined as the income received before any government intervention. It includes gross labor earnings and capital income, auto-consumption, imputed rent from owner-occupied housing, private transfers and contributory pensions paid by the social security system.

Net market income is market income minus direct taxes. As the ECH reports disposable income by source, estimation of direct taxes and contributions is done on the basis of legal schedules, assuming that taxes and contributions were entirely paid by workers. No taxes or contributions are assigned when workers worked informally.

Net market income plus direct transfers yields disposable income.^2^ Direct transfers include in-cash public transfers and food public transfers. In this study we classify four types of transfers: family allowances (conditional cash transfers – CCT), noncontributory pensions, food transfers and other direct trnasfers.

CCT is a means-tested program in which the beneficiaries are children under nineteen years of age. The transfer is conditional on school attendance and periodic health checkups. It is higher for secondary than primary students and the total household benefit increases at a decreasing rate with the number of siblings.

The non-contributory pension is a means-tested program directed at elderly adults (65 and over) who do not fulfill the requirements to obtain a contributory pension.

Food transfers are counted in terms of the monetary cost of three programs: free food hampers, free hot meals and a voucher that can be used to obtain food and hygiene products free of charge. All of these programs are targeted at vulnerable populations, including considerations such as income or illness (AIDS, tuberculosis, diabetes and lead poisoning, among others).

Other direct transfers include several programs directed at social security system contributors. These programs include unemployment insurance, maternity benefits, disability coverage and sickness allowances.

Post-fiscal income is disposable income minus indirect taxes. As the ECH does not report spending, a matching survey technique was used to take advantage of the information provided by an expenditure survey collected by the INE (2006) between November 2005 and October 2006.^3^ Indirect taxes are estimated using the legal schedule and assuming no tax evasion.

Final income is post-fiscal income plus in-kind education and health transfers. We need to solve two issues: how to estimate the monetary value of the services and how to allocate them across the population. To solve the first problem we use a cost of production approach: the transfers of educational and health services are equal to the per beneficiary public spending, estimated on the basis of administrative data. This approach has been criticized because it does not necessarily reflect the value of the service for the individual. From an economic perspective, we may argue that individuals would prefer to receive the equivalent amount of the in-kind benefit in money. This method also assumes that in-kind transfers are as valuable to the rich as to the poor, but we may expect that their value is lower for the poor, who would prefer in-cash transfers. Finally, cost of production does not take into account quality, efficiency or productivity. Consequently, it is likely to have a pro-rich bias because people with higher incomes tend to live in areas with higher-quality public services. However, other methods are similarly controversial and most of the literature uses the cost of production method (Verbist et al. 2012).

As to the second issue, there are two methods of assigning benefits: a) to the individuals who are actually using the services (consumption approach) or b) to the individuals who fulfill the requirements to use them in case of need (insurance value approach). We use the first approach to allocate the value of educational services and the second one to allocate health care benefits. These options are standard in the literature, although some studies prefer to use the consumption approach for both services (Verbist et al. 2012). In our case, we do not have information about the use of health care services.

Educational transfers are calculated using six separate levels: childcare, pre-school and primary school, middle school, high school, technical secondary school and tertiary education. The per beneficiary cost of each level is assigned to the students in the ECH. The highest transfer corresponds to tertiary education programs and the lowest to child care and primary level programs.

Public spending on health care occurs through two programs: the National Health Fund (NHF) and direct public health care provision for poor people. Beneficiaries of the NHF use a health care institution which in turn receives a transfer that varies with the age and sex of the beneficiary. The health care institutions supported by the NHF may be classified into three groups: private enterprizes in the mutual system, private insurance companies and public institutions. These public institutions also provide free health care to poor people. Therefore, public health care institutions are funded both by the NHF and the general public budget.

We use administrative data to calculate three different health care transfers: a) the per beneficiary transfer made to all private enterprizes in the mutual system; b) the per beneficiary transfer made to all private insurance companies; and c) the per beneficiary transfer to all individuals who use a public health care provider (regardless of whether funded by the NHF or general public budget). These transfers are imputed to the beneficiaries identified in the ECH. Note that we do not take into account differences in the cost of production by age or sex.

The largest transfer corresponds to individuals who use the public system, because the per beneficiary cost implicit in the general public budget is higher than the per beneficiary subsidy provided by the NHF. The estimated transfer in the private insurance system is lower than in the mutual heath care system, most likely because of the different demographic characteristics of the beneficiaries.

Note that in-kind transfers are measured by their per beneficiary budgetary cost. As other income sources are those reported by individuals in the ECH, we scale up all income sources except in-kind transfers to their macroeconomic values. We use scaled-up income for the study of average gaps and distribution. However, we do not use scaled-up values for the analysis of poverty and mobility (Section 4.2) because poverty lines are conceived for nonscaled values. Income was deflated by the Consumer Price Index (INE 2014).^4^

### 2.2 Poverty lines

We use three thresholds for measuring poverty. Extreme poverty corresponds to households whose per capita income (expressed in 2009 average domestic prices) was lower than US$ 2.5 PPP (2005) per day. For moderate poverty, we use a line of US$ 4 PPP (2005) per day. We convert these two thresholds to domestic 2009 prices by multiplying the PPP conversion factor for private consumption provided by World Bank (2016) by the ratio of the 2005 average consumer price index in Uruguay to the 2009 average consumer price index provided by the INE (2014). Per capita income of the households was also expressed in domestic 2009 average prices using the consumer price index.

We also work with the threshold used in Uruguay to provide the official measurement of poverty. This line incorporates an adult equivalent scale and varies by region. It was estimated in 2006 by the INE and the methodology and information used to update the line to 2009 prices are published in INE (2010). In 2009, the average national poverty line for all individuals was equal to US$ 7 PPP per capita per day.

### 2.3 Classification by race

The ECH asks individuals to identify their ethno-racial background in two distinct ways. First, individuals are asked, through separate questions, if they believe that they have African, Asian, white, indigenous, or other (can be specified) ancestry. This is followed by a question asking individuals to self-identify which of the previous ethnoracial groups they believe is their principal heritage. For this paper, we choose to classify the population according to self-identification of an individual’s principal heritage. We opted to use this identification method because it can be used to classify people with multiple ancestries. The sample is comprised of 4,236 Afro-descendants, 124,394 whites, 1,327 indigenous people and 97 cases with other or missing identification. Note that individuals of different ethno-racial identification may co-habit in the same household and share the same per capita income, taxes and benefits.

Table 1 reports the ethno-racial composition of our dataset as well as of the 2011 National Census, using main ethno-racial identification and also allowing for more than one ancestry. In both sources and for both questions, the majority of the population self-identifies as having white ancestry. Using the multiple-descendant criterion, in both datasets 5% of the population identifies as being of indigenous descent and between 8% and 9% report being of African descent.

**Table 1:** Racial classification by self-reported descent. Percentage

	CEQ database		Census	
	Allows multiple descent	Main racial descent	Allows multiple descent	Main racial descent
Total	113.6	100.0	107.7	100.0
Afro	9.3	3.4	8.1	4.8
	(9.03;9.64)	(3.22;3.57)		
White	98.9	95.5	93.9	92.3
	(98.74;98.94)	(95.31;95.70)		
Indigenous	5.1	1.0	5.1	2.5
	(4.88; 5.33)	(0.95; 1.12)		
Other	0.3	0.1	0.7	0.4
	(0.28; 0.38)	(0.05; 0.10)		

Source: *Censo de Población* 2011, INE (2011) and author’s calculations based on *Encuesta Continua de Hogares*, INE (2009).

a. 95% confidence intervals in brackets.

When using primary self-identification, in our dataset 3.4% of individuals self-identify as Afro-descendant, 95.5% as white and 1.0% as indigenous.^5^ The proportion of individuals identifying as predominantly Afro-descendant or indigenous is higher in the Census, which reports that 4.8% of the population is Afro-descendant and 2.5% is indigenous. The reason for the differences between the Census and the ECH are not certain, although Cabella and Nathan (2014) point out that during the Census, organizations representing Afro-descendants and indigenous people held campaigns to ensure that they were accounted for in official records, which would affect only the Census and not the subsequent ECH.

In Table 2 we show the composition of the population by age group and race. We observe that among Afro-descendants the share of children is higher and the share of elderly lower than among whites. Differences in fertility and mortality rates by race may explain these features (Cabella and Nathan 2014). Data in Table 2 also show that the working age share of the indigenous population is relatively higher. There are no previous studies that help explain this, although two possible causes may be given. One possibility is that working age indigenous individuals are immigrating to Uruguay, and another relates to cultural change in new generations questioning the traditional image of the society as exclusively of European ancestry. Encouragement of the acknowledgement of the indigenous contribution to Uruguayan identity may affect the self-identification of working age people (Cabella and Porzecanski 2015).

**Table 2:** Composition of population by age group and race. Percentage

Age group	White	Afro	Indigenous	
0 to 5	5.1	4.8	2.4*** †††
6 to 12	13.9	16.3***	10.0*** †††
13 to 17	8.4	9.9***	7.2 †††
18 to 24	9.9	9.6	9.2
25 to 64	47.7	49.9***	60.3*** †††
65 +	15.0	9.4***	11.0***
Total	100.0	100.0	100.0

Source: Author’s calculations based on CEQ database.

a. *** p<0.01, ** p<0.05, * p<0.1 for a test of proportions testing the null hypothesis that the composition of the population is equal between the column-race group and whites.

b. †††p<0.01, †† p<0.05, † p<0.1 for a test of proportions testing the null hypothesis that the composition of the population is equal between the Afro-descendants and indigenous people.

## 3 An overall description of welfare by group using disposable income

On average, the disposable income of indigenous people in Uruguay is 27% lower than that of whites. The ethno-racial gap is even larger for Afro-descendants, whose disposable income is 41% lower than that of whites. Minority groups are consistently over-represented in the lowest deciles of the disposable income distribution. Their share decreases sharply by decile, and they are underrepresented among the richest 10 percent (Table 3).

**Table 3:** Ethno-racial group structure within selected deciles of disposable income. Percentage

Decile	Whites	Afro	Indigenous	Total
1	91.1 (90.05;92.02)	7.6 (6.68;8.54)	1.3 (1.04;1.70)	100.0
2	92.3 (91.33;93.17)	6.4 (5.56;7.32)	1.2 (0.98;1.52)	100.0
5	95.6 (94.99;96.17)	3.1 (2.64;3.62)	1.2 (0.91;1.60)	100.0
9	97.9 (97.53;98.24)	1.3 (1.06;1.60)	0.7 (0.49;0.87)	100.0
10	98.7 (98.41;98.93)	0.6 (0.49;0.85)	0.5 (0.38;0.67)	100.0
All population	95.5 (95.31;95.70)	3.4 (3.22;3.57)	1.0 (0.95;1.12)

Source: Author’s calculations based on CEQ database.

a. 95% confidence intervls in brackets.

b. “other individuals” omitted.

**Fig. 1:**
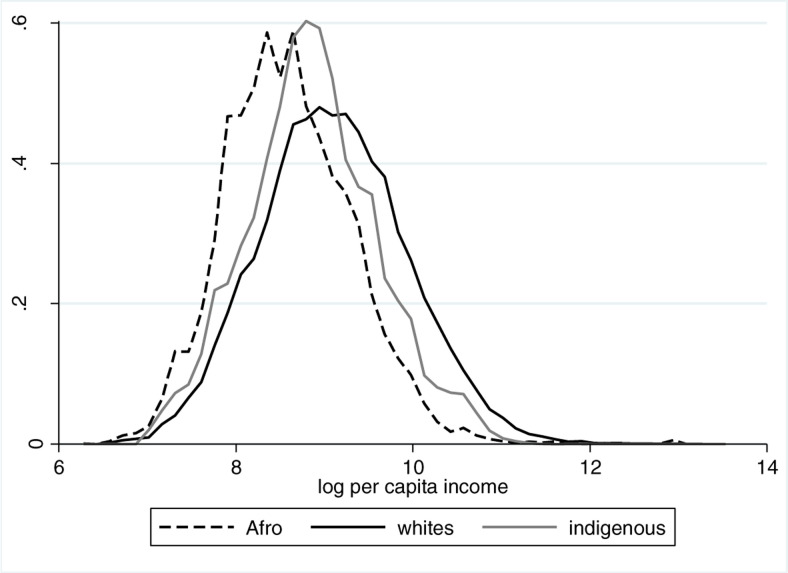
Density function of (log) disposable income for ethno-racial groups. Uruguay, 2009. Source: Authors’ calculations based on CEQ database

This pattern is reflected in Fig. 1, where we show the income distributions through kernel density functions of the (log) per capita household disposable income for whites, Afro-descendants and indigenous people, each shown separately. The majority of the Afro-descendant population is in the lowest income levels. On the other extreme, the income distribution of the white population is situated to the far right, revealing that the majority of white Uruguayans have higher levels of income than indigenous people and Afro-descendants. The overall picture shows that whites are the most advantaged ethno-racial group while Afro-descendants are the most disadvantaged.

Little is known about indigenous people in Uruguay, probably because they are a small percentage of the population. Thus, we do not have rigorous explanations of the reasons that they experience intermediate levels of welfare between those of Afro-descendants and whites. A number of explanations can be suggested regarding their better situation relative to Afro-descendants. It is possible that in Uruguay, prejudice against Afro populations is deeper than prejudice toward indigenous people, which would be reflected in income differences. This may be reinforced if the construction of identity is different between Afro and indigenous individuals. Indeed, as we have already mentioned, the rise of social legitimacy of indigenous history has led some persons who are visibly of primarily European ancestry to self-identify as indigenous. Another argument is the difference in educational levels between groups: the average years of schooling among the population aged 25 or over is lower among Afro-descendants than for indigenous people and whites (by around two years), whereas there are no statistically significant differences between indigenous and white populations.

Inequality within each group, as measured by the Gini and Theil index, is presented in Table 4. Consistent with data in Fig. 1, inequality within the indigenous group is lower than that within the white population. When comparing Afro-descendants and whites, both indices show that the racial differences in inequality are not statistically significant. Further analysis of the microdata reveals that a small proportion of the Afro-descendant population does very well, and falls within the top 0.05% of the overall income distribution. If we do not include this richest segment of the population, disposable income inequality among whites becomes significantly higher than that among Afro-descendants when measured by either index.

**Table 4:** Gini index and poverty headcount ratio by race and difference between races (disposable income). Percentages

	Gini index	Theil index	US$ 2.50 PPP per day	US$4 PPP per day	National poverty line
All	45.8	39.1	2.0	8.4	22.3
Whites	45.6	38.6	1.9	8.0	21.3
Afros	44.3	48.1	5.0	19.1	47.5
Indigenous	40.6	28.6	3.0	11.2	30.8
Afro-white diff.	−1.3	9.5	3.1***	11.1***	26.2***
Indigenous-white diff.	4.9***	−10.0***	1.1	3.2**	9.4***
Afro-indigenous diff.	3.6	19.5	2.0*	7.9***	16.7***

Source: Author’s calculations based on CEQ database.

a. Differences between Gini index and poverty rate are in percentage points.

b. *** p<0.01, ** p<0.05, * p<0.1 for a test of means testing the null hypothesis that the column-index is equal between groups.

In Table 4 we also present poverty rates by ethno-racial group. Afro-descendants face the highest incidence of extreme poverty (US$ 2.50 PPP per day) at 5%, followed by indigenous individuals (3%) and white Uruguayans (1.9%). At higher poverty thresholds, differences between racial groupings are higher: according to the national line, the poverty rates faced by Afro-descendants are twice that of whites, whereas indigenous people are 40% more likely than whites to be poor.

In short, indigenous people are the most homogenous group and are situated in an intermediate position between Afro-descendants and whites. As for African descendants, with the exception of a small portion who are very wealthy, they face the deepest income constraints.

## 4 Effects of the tax and transfer system

To determine if a policy is effective at reducing inequalities in ethno-racial terms, we perform a racial incidence analysis in Section 4.1. We apply definitions of progressivity of programs and taxes in an ethno-racial space, following Lustig (2014b). A program is considered to be progressive (regressive) in ethno-racial terms if those groups who face lower (greater) incomes receive a greater share of resources than their share of market income. Conversely, a tax is considered progressive (regressive) if the amount paid by the disadvantaged group is lower (higher) than their share of market income. Both taxes and transfers are considered neutral if the incidence of spending is similar to the share of market income.

The progressivity of a program may be due in part to the number of individuals from a specific group receiving the program for reasons other than their race or ethnicity. For example, a program targeted at individuals living in poverty should disproportionately reach groups that have more individuals in poverty. Thus, examining the effect of public policies on poverty exit across ethno-racial groups is an important element in determining whether a program is prodisadvantaged group. This is the topic of Section 4.2.

We complement these analyzes by assessing the contribution of public programs to narrowing the average ethno-racial gaps. The final effect is a combination of the benefit amount of each program and the extent to which each group is represented in the target population of the programs, which in turn is related to the groups’ income and demographic characteristics, among other factors. Although it is not within the scope of this paper to disentangle the sources of public program effects, in Section 4.3 we perform an analysis of the average ethno-racial gaps which explores some of these issues.

It is necessary to note some caveats about the methodology. One is related to the consideration of benefits only in monetary terms. The reduction of inequalities in terms of resources is not a measure of the reduction of disparities that has been achieved. For example, Afro-descendants may receive a greater share of educational resources, but this could be insufficient to reduce the ethno-racial gap in educational achievement. Besides, and related to this issue, the quantity of resources is not a guarantee of quality.

Another caveat is related to the “accounting” method, which does not account for the effects of potential behavioral responses, general equilibrium issues and intertemporal questions. Fiscal policy encourages behavioral responses that have been proven to exist in Uruguay. For example, Bergolo and Cruces (2014, 2016) find that increases in benefits from contributory and assistance programs produce complex patterns of responses including changes in labor participation, changes in registered employment and underreporting of taxable income. However, the “accounting” methods used in this work are considered efficient for assessing who benefits from public transfers and who bears the burden of taxes, and have the virtue of simplicity and transparency, especially when focusing on average incidence.

### 4.1 A racial incidence analysis

The incidence of different government interventions as a share of the program’s budget across ethno-racial groups is presented in Table 5. As the incidence of direct and indirect taxes across all ethnoracial groups is very similar to the share of market income held by the groups, the overall impact of direct taxation is neutral in ethno-racial terms.

**Table 5:** Ethno-racial incidence of fiscal intervention. Percentage of national values

	White	Afro-descendant	Indigenous
Population	95.5	3.4	1.0
Market income	97.2	1.9	0.7
Direct taxes	97.5	1.8	0.6
All direct transfers	92.9	5.7	1.3
CCT	92.7	6.1	1.3
Non-contributory pension	91.9	6.7	1.4
Food transfers	90.6	7.9	1.5
Other direct transfers	95.4	3.3	1.2
Indirect taxes	97.2	1.9	0.8
All in-kind transfers	95.0	3.8	1.1
Education	95.1	3.7	1.1
Health	94.9	3.9	1.1

Source: Author’s calculations based on CEQ database

The incidence of direct transfers is highly progressive, with Afro-descendants and indigenous people receiving 5.7% and 1.3% of national direct transfers, respectively, compared to the 1.9% and 0.7% of market income held by these respective groups. The progressivity is particularly robust in food transfers, which are 21% of direct transfers. CCT expenditures (31% of direct transfers) and non-contributory pensions (19%) are also progressive, whereas other direct transfers (29%) are neutral.

Finally, in-kind transfers are progressive: Afro-descendants receive 3.7% of educational spending and indigenous people, 1.1%; the shares of health care are 3.9% for Afro-descendants and 1.1% for indigenous people.

### 4.2 Poverty and mobility: From market to disposable income

Three well-known FGT poverty measures calculated using market and disposable income are presented in Table 6. Before fiscal policy, Afro-descendants are the poorest group in terms of poverty incidence, gap and severity. They are followed by the indigenous people and finally, whites.

Direct taxes and transfers strongly reduce both extreme and moderate poverty measured by the three indices. Poverty measured using the national threshold also declines, but the reduction is not as large. All ethnoracial groups benefit from these reductions, but the gaps persist.

**Table 6:** Poverty by race. Percentage

	Total	Whites	Afro	Indigenous	
FGT(0)					
*Poverty line: $2.50 PPP/day*					
Market income	5.9	5.6	14.9***	7.7*	†††
Disposable income	2.0	1.9	5.0***	3.0	†
*Poverty line: $4 PPP/day*					
Market income	12.9	12.3	28.5***	16.3**	†††
Disposable income	8.4	8.0	19.1***	11.2**	†††
*National poverty line*					
Market income	25.3	24.2	52.1***	35.4***	†††
Disposable income	22.3	21.3	47.5***	30.8***	†††
FGT(1)					
*Poverty line: $2.50 PPP/day*					
Market income	2.1	2.0	5.5***	2.5	†††
Disposable income	0.4	0.4	0.9***	0.4	††
*Poverty line: $4 PPP/day*					
Market income	4.8	4.6	11.6***	6.3**	†††
Disposable income	2.1	2.0	4.5**	2.7*	†††
*National poverty line*					
Market income	10.2	9.6	23.8***	14.1***	†††
Disposable income	7.0	6.7	16.4***	9.9***	†††
FGT(2)					
*Poverty line: $2.50 PPP/day*					
Market income	1.1	1.0	2.9***	1.2	†††
Disposable income	0.1	0.1	0.3**	0.1	††
*Poverty line: $4 PPP/day*					
Market income	2.6	2.4	6.4***	3.2	†††
Disposable income	0.8	0.7	1.7***	0.9	†††
*National poverty line*					
Market income	5.6	5.3	14.0***	7.9***	†††
Disposable income	3.1	2.9	7.7***	4.6***	†††

Source: Author’s calculations based on CEQ database.

a. *** p<0.01, ** p<0.05, * p<0.1 for a test of means testing the null hypothesis that the column-group is equal to whites.

b. ^†††^p<0.01, ^††^, p<0.05, ^†^ p<0.1 for a test of means testing the null hypothesis that Afro-descendants is equal to indigenous people.

To determine if a policy is pro-disadvantaged group, we use the concept of fiscal mobility, which refers to movements across the income distribution due to fiscal policy (Lustig 2014b). Following this idea, we calculate the probabilities of exiting poverty through fiscal intervention, and more generally the rates of transition between socio-economic classes.

To determine if a program is prodisadvantaged group, we consider whether it equalizes the poverty rates of ethno-racial groups, i.e. whether it leads to the ethno-racial composition of the poor being more representative of the population as a whole. In order to achieve this goal, fiscal policy should treat individuals of different races or ethnicities differently. In terms of probabilities of climbing out of poverty, the policy is considered successful if the rate is higher for the pre-policy disadvantaged ethno-racial group(s) than it is for the advantaged group(s) (see Appendix).

The population is divided into five income classes: the extreme poor (y<US$ 2.50 PPP per day), the moderate but not extreme poor (US$ 2.50 PPP≤y<US$4 PPP), the vulnerable class (US$ 4≤y<US$10 PPP), the middle class (US$ 10≤y<US$50 PPP) and finally, the rich (y≥US$ 50 PPP).

These classes are defined for both market and disposable income. We denote the classes by *c*, where *c* ranges from 1 (the poorest) to 5 (the richest). The probability of upward mobility for each class *c* is:

(1) $$H_{c}^{up}=\frac{1}{n_{c}^{m}}\sum_{i=c+1}^{5}n_{i}^{d}\;\mathrm{for}\;c=1,2,3,4$$

where $n_{c}^{m}$ is the number of individuals in class c according to market income and $n_{i}^{d}$ is the number of individuals in class i according to disposable income. Similarly, the probability of downward mobility is calculated as:

(2) $$H_{c}^{down}=\frac{1}{n_{c}^{m}}\sum_{i=1}^{c-1}n_{i}^{d}\;\mathrm{for}\;c=2,3,4,5$$

In Table 7 we present the probabilities of moving between income classes for each ethno-racial group and their 95% confidence intervals. Across all ethno-racial groups, the probability that an individual exits extreme poverty as a result of government intervention is over 60%, while the probability of escaping moderate poverty is more than 50%. Overall, the probability of exiting poverty (both extreme and moderate) is about 35%. There are no statistically significant differences between ethnoracial groups, so the impact of direct taxes and of transfers does not contribute to narrowing ethno-racial gaps in terms of poverty.

**Table 7:** Probabilities of exiting market income classes. Percentages

Class defined by market income	Whites		Afro		Indigenous	
	H^up^	H^down^	H^up^	H^down^	H^up^	H^down^
Extreme poor	66.3		66.6		61.7	
	(63.37;69.18)		(58.38;73.98)		(45.31;75.82)	
Moderate poor	54.4	0.1	53.3	0.0	51.7	0.0
	(51.70; 57.07)	(0.01; 0.64)	(45.22; 61.23)	(0.00; 0.53)	(37.10; 66.07)	(0.00; 2.22)
Poor (all above)	35.7		33.2		32.5	
	(33.75;37.60)		(27.98; 38.91)		(23.63; 42.80)	
Vulnerable class	4.2	0.1	3.0	0.1	6.1	0.4
	(3.80; 4.66)	(0.03; 0.17)	(1.92; 4.58)	(0.01; 0.67)	(3.76; 9.90)	(0.06; 2.90)
Middle class	0.1	1.1	0.1	2.9	0.0	0.6
	(0.03; 0.10)	(0.98; 1.29)	(0.01; 0.37)	(1.67; 5.02)	(0.00; 0.56)	(0.17; 1.88)
Rich		17.4		13.8		20.2
		(15.85; 19.15)		(5.95; 28.72)		(8.88; 39.58)

Source: Author’s calculations based on CEQ database.

a. 95% confidence intervals in brackets

The socio-economic classes above the poverty line have less mobility. The probability that an individual in the vulnerable class will enter the middle class is very low, ranging from 3% for Afro-Uruguayans to 6.1% for indigenous people. The probability of moving from the middle class into the richest class is almost nil for all races. Finally, direct taxes and transfers have a negligible effect on downward mobility, except among the rich.

### 4.3 Average gaps: From market to final income

The average income of Afro-descendants and indigenous people relative to the white population, across all income concepts, is presented in Table 8. Before fiscal policy, the per capita income of Afro-descendants relative to whites is 0.559 and the ratio for indigenous people is 0.708.

#### 4.3.1 Effect of taxes

Direct taxes do not affect the afro-white income gap, but they reduce the indigenous-white gap, as can be seen in the comparison between market and net market income (Table 8). Indeed, the effective direct tax rates are 6.1% for whites, 5.5% for Afro-descendants and 5.0% for indigenous people. Part of the explanation for these differences is related to the higher market income level of whites and the higher weight of non-taxable income sources for indigenous individuals relative to Afro-descendants.

**Table 8:** Per capita income of Afro-descendants and indigenous people relative to the per capita income of whites

Income concept	Afro	Indigenous
Market income	0.559 (0.557;0.562)	0.708 (0.706;0.709)
Net market income	0.563 (0.560;0.565)	0.716 (0.714;0.717)
Net market income + CCT	0.569 (0.567;0.572)	0.719 (0.717;0.720)
Non-contributory pensions	0.572 (0.570;0.574)	0.720 (0.718;0.721)
Food transfers	0.572 (0.570;0.574)	0.720 (0.718;0.721)
Other direct transfers	0.566 (0.563;0.568)	0.719 (0.718;0.720)
Disposable income	0.590 (0.588;0.593)	0.730 (0.728;0.731)
Post-fiscal income	0.593 (0.591;0.595)	0.730 (0.728;0.731)
Post-fiscal income + Health	0.631 (0.629;0.633)	0.755 (0.754;0.756)
Child care and primary education	0.619 (0.617;0.621)	0.744 (0.743;0.745)
Secondary education	0.601 (0.599;0.603)	0.736 (0.735;0.737)
Tertiary education	0.589 (0.587;0.591)	0.727 (0.726;0.729)
All education	0.622 (0.620;0.624)	0.748 (0.747;0.749)
Final income	0.656 (0.654;0.658)	0.771 (0.770;0.772)

Source: Author’s calculations based on CEQ database.

a. 95% confidence intervals (in brackets) estimated by bootstrapping techniques

Indirect taxes do not affect ethnic-racial gaps when comparing disposable and post-fiscal income.

#### 4.3.2 Effect of direct transfers

The ethno-racial gap decreases as a result of direct transfers (the change from net market to disposable income). Mean income of Afro-descendants relative to whites grows from 0.563 to 0.590 and in the case of indigenous people, it rises from 0.716 to 0.730.

The gap-reducing effect of direct transfers is certainly explained by the overrepresentation of Afro-descendants and indigenous people among the poorest populations. In Table 9 we report the share of ethno-racial groups in the population as a whole and in the first decile of the net market income distribution. We can see that Afro-descendants and indigenous people are overrepresented in the first decile. Although it is not possible to identify the potential beneficiaries of direct transfers in the database, the population in the first decile is undoubtedly part of the target population of most direct transfers. The overrepresentation of minorities in the first decile is also observed when we consider only households with children and households with members aged 65 and over, which are the potential beneficiaries of two programs (CCT and non-contributory pensions).

**Table 9:** Distribution of ethno-racial groups in the population and in the first decile of net mket income distribution. Percentages

	Whole population			First decile of net market income		
	Whites	Afro	Indigenous	Whites	Afro	Indigenous
Whole population	95.5 (95.31;95.70)	3.4 (3.22;3.57)	1.0 (0.95;1.12)	90.8 (89.76;91.78)	7.8 (6.89;8.81)	1.3 (1.06;1.72)
Population living in households with children	94.8 (94.49;95.06)	4.0 (3.79;4.31)	1.1 (0.99;1.23)	90.7 (89.60;91.73)	7.9 (6.90;8.92)	1.4 (1.08;1.78)
Population living in households with elderly members	96.6 (96.27;96.89)	2.6 (2.34;2.90)	0.7 (0.63;0.88)	91.1 (88.53;93.21)	8.2 (6.14;10.75)	0.5 (0.27;1.10)

Source: Author’s calculations based on CEQ database.

a. 95% confidence interval (in brackets).

b. “other individuals” omitted

However, there are factors other than income differences between groups that may contribute to the programs’ closing of the ethno-racial gap.

Coverage of two programs is affected by age distribution: groups with more children are more likely to receive CCT whereas groups with more elderly individuals are more likely to receive non-contributory pensions. However, some indicators suggest that the effect of age structure may be not important. As mentioned in Section 2.3, Afro-descendants have more children and fewer 65+ persons than whites: this is captured by the population share living in households with children and in households with elderly members, as presented in Table 10. But these shares are not different when focusing on people in the first decile of net market income. In the case of indigenous people, there are fewer households both with children and with elderly than among whites (Section 2.3). In Table 10 we observe that although the share of households with children is higher in this group than among whites, the opposite holds in the case of households with elderly members (in the whole population and in the first decile).

**Table 10:** Coverage (in percentages) and per capita benefit (in Uruguayan pesos) of direct transfers

	Whole population			First decile of net market income		
	Whites	Afro	Indigenous	Whites	Afro	Indigenous
Share of population living in households with children	62.6	75.3***	67.3*** †††	93.4	94.1	96.3*
Share of population living in households with elderly members	25.4	19.3***	18.1***	12.1	12.6	4.8*** †††
Program coverages						
CCT	19.5	40.8***	27.7*** †††	74.5	76.9	71.1
*among households with children*	*30.9*	53.8***	40.8***†††	*79.8*	*81.7*	*73.8*
Non-contributory pensions	4.7	9.2***	6.1††	16.7	20.8	12.7 †
*among households with elderly members*	*10.1*	23.9***	17.7**	*67.2*	*64.8*	*54.9*
Food transfers	20.4	43.9***	32.4*** †††	76.7	84.8***	81.9
Other direct transfers	16.7	16.9	17.0	12.9	13.5	11.9
All direct transfers	40.8	65.2***	54.0*** †††	94.5	95.5	98.0***
Per capita benefit among beneficiaries							
CCT	288	281	282	296	289	298
Non-contributory pensions	2265	2212	2204	2503	2294	3613
Food transfers	304	347***	292 †††	392	435**	374 ††
Other direct transfers	543	521	614	721	749	1332
All direct transfers	654	700*	642	865	920	787

Source: Author’s calculations based on CEQ database.

a. *** p<0.01, ** p<0.05, * p<0.1 for a test of proportions (share and coverage) / test of means (per capita benefit) testing the null hypothesis that the column-group is equal to whites

b.††† p<0.01, †† p<0.05, † p<0.1 for a test of proportions (share and coverage) / test of means (per capita benefit) testing the null hypothesis that the Afro-descendants column is equal to that of indigenous people.

c. Children are those aged 18 and under and elderly are those aged 65 and over.

d. Beneficiaries are all household members in beneficiary households

Coverage is also affected by program uptake and eligibility requirements other than income and age, but we do not have information to assess their importance. The similar coverage by race for CCT and non-contributory pension within the first decile suggests that their role is small. However, in a study of a 2005–2007 assistance program, Burdin and de Melo (2009) show that households headed by an Afro-descendant were more likely to be covered than other households with similar characteristics, particularly income. Also, there is evidence that at present, minority workers are less likely to contribute to the social security system than whites (Bucheli and Cabella 2010), which may explain why minority elders are more likely to be covered by non-contributory pensions.

With respect to food transfers, coverage is more likely among Afro-descendants than whites both in the first decile and across the whole population. This may be related to differences in eligibility requirements such as nutritional deficiency or illness incidence. The average food transfer is also different between groups: once again, Afro-descendants benefit more than whites.

The effect of the addition of all programs (all direct transfers) is mediated by two important factors. One is the likelihood of being covered by more than one program: multiple coverage is more likely among indigenous individuals than whites (Table 10). The other is the substantial difference in the benefit amounts of the programs. This factor is probably not very important because ethno-racial age structure and coverage rates in the first decile are similar between groups.

In sum, the fact that the income of Afro-descendants and indigenous people is lower than that of whites appears to be the most important factor in explaining the gap-reducing effect of direct transfers.

#### 4.3.3 Effect of in-kind transfers

The shift from post-fiscal to final income, which takes into account the impact of in-kind benefits, reduces the ethno-racial gap by more than direct transfers do (Table 8). The income ratio between Afro-descendants and whites grows from 0.593 to 0.656 and the ratio between indigenous people and whites increases from 0.730 to 0.771. Both health and educational transfers contribute to this narrowing.

The effect of health transfers comes from both the amount and coverage.

As described in Section 2.1, there are four health care possibilities which involve public funding: direct public health care provision and three options under the NHF, namely public institutions, the mutual system and the private insurance system. In Table 11 we show the coverage of each option and the transfer amount imputed by this study. Afro-descendants and indigenous people are more likely than whites to be covered by direct public health care provision, which is the option with the highest transfer. This is not surprising given that Afro-descendants and indigenous people are more concentrated in the poor segments of society than whites. Also, among those who are beneficiaries of the NHF, 10.5% of Afro-descendants compared to 6.6% of whites opt for public institutions (the difference is statistically significant), which may be explained by the fact that copayments and other out-of-pocket costs are higher in the private system. Thus, in monetary terms, the ethno-racial gap narrows.

**Table 11:** Coverage of health transfers (percentage) and per capita benefit (Uruguayan pesos)

	Whites	Afro	Indigenous	Benefit
Coverage by health transfers	81.8	91.3***	88.1*** †††	
Direct public health care	35.4	57.0***	45.6*** †††	1141
National Health Fund	46.4	34.3***	42.5* †††	
Private insurance company	0.9	0.1***	0.5*	715
Mutual health institution	42.5	30.6***	38.5** †††	968
Public institution	3.0	3.6	3.6	1141

Source: Author’s calculations based on CEQ database.

a. ***p<0.01, ** p<0.05, *p<0.1 for a test of proportions testing the null hypothesis that the column-group is equal to whites.

b. ††† p<0.01, †† p<0.05, † p<0.1 for a test of proportions testing the null hypothesis that Afro-descendants is equal to indigenous people.

c. The benefit is the estimated average per beneficiary public cost

We are aware, however, that our estimate of benefits only takes into account the amount of public funding, which says nothing about the quality differences among the options. There is not a set of indicators for quality or efficiency. The Ministry of Health (Ministerio de Salud Pública 2014) suggests that the average wait time for patients to see specialists is similar between public and private institutions, and for some specialties is even shorter in public than in private institutions. This indicator also varies between the capital and the rest of the country, suggesting that there is heterogeneity within sectors. However, the lack of research on quality and efficiency in the Uruguayan health system makes it impossible to assess whether differences in transfers accurately reflect differences in services.

In turn, the effect of educational transfers varies by education level. Child care and primary education have the strongest effect on narrowing the average ethno-racial gaps; secondary education also contributes to narrowing the gaps, but tertiary education does not have statistically significant effects (Table 8). The factor explaining these patterns is the ethno-racial differences in public education coverage.

Public education coverage depends on total enrollment and on how many opt for the public system. In Table 12 we report the public education coverage rate, the overall enrollment rate and the percentage of enrolled students who chose a public school, each by race and age group. Each age group corresponds roughly to a level of education. The over-age incidence rises with age, and is very high in the 18-24 age-group.

**Table 12:** Rates of coverage, enrollment and choice of public option among enrolled, by age group and race. Percentage

	Whites	Afro	Indigenous
Public coverage			
42 to 12	72.8	85.4***	87.0***
13 to 17	70.1	71.0	71.4
18 to 24	33.8	18.5***	19.8***
Enrollment			
4 to 12	97.4	97.5	99.6***†††
13 to 17	84.0	76.5***	76.4
18 to 24	40.1	19.1***	20.8***
Public option of enrolled			
4 to 12	74.7	87.7***	87.4***
13 to 17	83.4	92.9***	93.5***
18 to 24	84.3	96.9***	95.0**

Source: Author’s calculations based on CEQ database.

a. *** p<0.01, ** p<0.05, * p<0.1 for a test of proportions testing the null hypothesis that the column-group is equal to whites.

b. ††† p<0.01, †† p<0.05, † p<0.1 for a test of proportions testing the null hypothesis that Afro-descendants is equal to indigenous people

The rates reflect the fact that public education coverage is lower for whites than for other racial groups in the 4-to-12 age range. Child care and primary school attendance is nearly universal across ethno-racial lines. Thus, the differences in coverage are due to individual choices between public and private systems. Indeed, the richest households use the private school system, which has no public funds.

For children between 13 and 17 years of age, there are no statistical differences in coverage between racial groups. This is the result of opposing effects: whites have higher enrollment rates (although the difference with indigenous people is not statistically significant) but they are more likely to opt for the private system.

Finally, the coverage rate for the 18-24 age-group is notoriously lower for Afro-descendants and indigenous people than for whites. Once again enrollment and private/public choice have opposite effects. But for these ages the ethno-racial differences in school drop-out rates are very important, more than offsetting the effect of private/public choice. An important aspect of the 18-24 age range is that 22% of whites are attending tertiary level education, as compared to 5% of Afro-descendants and 9% of indigenous people. Most students at the university level use public services, and the highest imputed transfer is at this level of education. Thus, the ethno-racial differences in drop-out rates at the secondary level means that the minorities do not capture the actual and potential benefits of college attendance.

In sum, the main factor that explains the educational effect that closes the gap is the difference between races in choosing the public option. As with health, we are not taking into account the differences in quality between public and private education and among public establishments. But in the case of education, many factors suggest that these differences lead to an overestimation of the racial gap closure. Indeed, in addition to the relative low quality of the average public education, several studies point out that public educational services vary between geographical areas. Specifically, disadvantaged areas provide educational services with lower quality. This affects populations with fewer economic resources, although students may choose the school. However, the opportunities for choice are limited: Bogliaccini and Rodríguez (2015) indicate that most of the students attend an establishment near their home because of transportation costs.

One of the sources of quality differences within the public system is the mechanism of allocating teachers to schools: the administration makes an ordered list of teachers based on their curriculum and the best teachers get first choice in their school of preference. Bogliaccini and Rodríguez (2015) find that in both primary and secondary education, more experienced teachers are concentrated in advantaged socio-economic areas. In a study of primary level education, Luschei and Carnoy (2010) reinforce the importance of this finding, concluding that teacher experience is strongly related to student outcomes. Though there is a monetary incentive for primary-level teachers who choose schools in disadvantaged areas, its effect on choice is statistically significant but small, as found by Cabrera and Webbink (2016). These authors suggest that the non-pecuniary compensations outweigh the monetary incentives because teachers’ wages are low.

Another source of inequality within public supply comes from differences in availability of educational technology and materials across schools. A share of these inputs is funded by associations of students’ parents, which has a direct effect on these resources. In addition, Bogliaccini and Rodríguez (2015) argue that management abilities of school principals are better in high than in low socio-economic areas, a fact which allows them to capture more resources from the central administration.

## 5 Concluding remarks

In this study we present an incidence analysis of taxes and social spending and an assessment of their impact on poverty and average gaps between ethno-racial groups (Afro-descendants, indigenous people and whites) in Uruguay. Taxes are neutral in ethno-racial terms and do not affect poverty and average gaps between groups. Direct transfers are progressive in ethno-racial terms and contribute to reducing the average gaps. They reduce poverty across all ethno-racial groups, but they do not specifically favor those groups which face higher rates of poverty. In-kind transfers are also progressive, and their contribution to narrowing gaps is more important than that of direct transfers. Therefore, these results support the idea that fiscal policy reduces (but does not eliminate) the ethnic divide.

This conclusion contrasts with findings for Guatemala (Cabrera et al. 2015), Brazil (Pereira 2016) and Bolivia (Lustig 2014b) obtained using similar methodology. In those three cases, the impact of fiscal policy on ethnic divides is very small compared to its impact in the Uruguayan case. This is not related to the ethno-racial design of policies: Uruguayan programs are targeted on a race-blind basis. The difference comes from the fact that minority groups are more likely than whites to be poor before fiscal policy, and fiscal policy is more successful in reducing overall inequality and poverty in Uruguay than in Guatemala, Bolivia or Brazil. Indeed, the main reason for the reduction of the ethnic divide in Uruguay can be found in the low-income situation of Afro-descendants and indigenous people. Social spending is progressive for two reasons: program design ensures that the low-income population is the target of most direct transfers, and public services are chosen less often by those with higher incomes.

Two critical questions emerge from this study. First, the effect of in-kind transfers does not take into consideration potential quality differences between public and private systems. As the private system is more often chosen by those with higher incomes, we might suspect that quality is better in the private system. Thus, the estimated reduction of the ethno-racial gaps should be interpreted with caution. Second, enrollment rates decline with age more sharply for Afro-descendants and indigenous people than for the white population. Indeed, there are no attendance gaps at the primary level, but differences appear at the secondary level and are considerably higher at the tertiary level, as in most Latin American countries (Zapata et al. 2011; Busso et al. 2005; Marteleto et al. 2016; Tas et al. 2014). Thus, minorities are not capturing the full potential of education transfers. A relevant consequence of this fact is that the investment in human capital is lower for Afro-descendant and indigenous populations than for whites. This is particularly important given the low tertiary education graduation rates of minority groups. This phenomenon should be a special focus of policy.

## Footnotes

1 see: http://www.commitmentoequity.org/

2 Note that social security contributions are treated as savings and so are included in disposable income.

3 From the expenditure survey, we identify fifty-two consumption baskets. Each basket is composed of goods or services with high substitutability and with the same tax rate. For each basket we run Tobit models of household expenditures on a set of variables that are available in the expenditure survey and the ECH. Using the estimated coefficients we predict the consumption of each household in the ECH. The residuals are reallocated using the “uvis” command in STATA 12. Finally, we estimate the indirect taxes by applying the scheduled tax rate of each basket assuming no evasion.

4 There are different ways of dealing with information that combines monthly income reported in interviews in different months with yearly macroeconomic values. As similar results were obtained without deflation (inflation measured by the consumer price index was 5.9% in 2009) we did not deeply analyze other alternatives.

5 For our purposes, individuals who identified as “Asian” or “other” are both included in the category “other”.

## Open Access

This article is distributed under the terms of the Creative Commons Attribution 4.0 International License (http://creativecommons.org/licenses/by/4.0/), which permits unrestricted use, distribution, and reproduction in any medium, provided you give appropriate credit to the original author(s) and the source, provide a link to the Creative Commons license, and indicate if changes were made.

## Appendix: The poverty exit rate (hazard rate) and the poverty ratio

If the pre-policy poverty rate is higher for group *a* than for group *ω*, the post-fiscal poverty rate will be equal only if the hazard rate of exiting poverty is higher in group *a* than in group *ω*.

Suppose that in the pre-policy situation the poverty rate is higher for group *a* than for group *w*. If we denote the number of poor by 7 and the number of persons in each group by *N*:

$$\frac{Z_{a,pre}}{N_{a}}>\frac{Z_{w,pre}}{N_{w}}$$

The hazard rate of group *i* (*i = a, w*) for leaving poverty is *h_i_*:

$$Z_{i,pre}$$

So, a positive hazard rate indicates that the policy reduces the poverty rate.

If the post-policy poverty rates of the groups are equal:

$$\frac{Z_{a,pos}}{N_{a}}=\frac{Z_{w,pos}}{N_{w}}$$

We can rewrite the equality as:

$$\frac{Z_{a,pre}+\left(Z_{a,pos}-Z_{a,pre}\right)}{N_{a}}=\frac{Z_{w,pre}+\left(Z_{w,pos}-Z_{w,pre}\right)}{N_{w}}$$

$$\frac{Z_{a,pre}}{N_{a}}+\frac{\left(Z_{a,pos}-Z_{a,pre}\right)}{Z_{a,pre}}\frac{Z_{a,pre}}{N_{a}}=\frac{Z_{w,pre}}{N_{w}}+\frac{\left(Z_{w,pos}-Z_{w,pre}\right)}{Z_{w,pre}}\frac{Z_{w,pre}}{N_{w}}$$

$$\frac{Z_{a,pre}}{N_{a}}\left[1-h_{a}\right]=\frac{Z_{w,pre}}{N_{w}}\left[1-h_{w}\right]$$

$$\frac{\frac{Z_{a,pre}}{N_{a}}}{\frac{Z_{w,pre}}{N_{w}}}=\frac{\left[1-h_{w}\right]}{\left[1-h_{a}\right]}>1\Leftrightarrow h_{w}<h_{a}$$
